# Exploring challenges and facilitators to E-learning based Education of nursing students during Covid-19 pandemic: a qualitative study

**DOI:** 10.1186/s12912-023-01430-6

**Published:** 2023-08-22

**Authors:** Fereshteh Araghian Mojarad, Ali Hesamzadeh, Tahereh Yaghoubi

**Affiliations:** 1 Traditional and Complementary Medicine Research Center, Addiction Research Institute, Mazandaran University of Medical Sciences, Sari, Iran; 2 Behshahr Faculty of Nursing, Mazandaran University of Medical Sciences, Behshahr, Iran

**Keywords:** Challenges, Facilitators, e-learning, Nursing students, COVID-19

## Abstract

**Background:**

During the COVID-19 pandemic, there was a shift to e-learning and online education in educational and learning processes. Research has shown that nursing students who are satisfied with e-learning tend to have better learning outcomes. Therefore, this qualitative study aimed to explore the challenges and facilitators of e-learning for nursing students during the pandemic.

**Methods:**

This qualitative study utilized a content analysis approach. Sixteen participants with nursing education experience were purposively selected and interviewed using a semi-structured format. The data collected were analyzed using the conventional qualitative content analysis approach.

**Results:**

Through data analysis, two main categories were identified: e-learning challenges and facilitators. The e-learning challenges included subcategories such as inexperienced teachers, ineffective learning, academic cheating, system problems, and inappropriate evaluation. The facilitators included subcategories such as improving education, and promoting online exams.

**Conclusions:**

While e-learning was adopted as a substitute for in-person education during the COVID-19 pandemic, its implementation involves both challenges and facilitators. By addressing the challenges and optimizing the facilitators of e-learning, nursing schools can successfully adapt to this new mode of education in the post-pandemic era and provide high-quality education to their students.

**Supplementary Information:**

The online version contains supplementary material available at 10.1186/s12912-023-01430-6.

## Background

The COVID-19 pandemic has transformed the healthcare environment and the learning settings of nursing students, resulting in a shift from traditional face-to-face education classes to online courses. This shift has impacted students’ learning opportunities and has required nursing schools to adapt to new modes of education delivery [1]. The pandemic has forced universities around the world to rapidly improvise and adopt online teaching methods [2]. Consequently, nursing education underwent many changes, and nursing students inevitably turned to different types of e-learning through various social networks [3]. E-learning is the use of electronic resources like the internet, computers, and smartphones to acquire and disseminate knowledge [4]. Iran is among the countries that have adopted various e-learning methods as an alternative to traditional education [5].

Given the rapid changes in teaching-learning environments, e-learning based education is an alternative or supplement to conventional teaching and it offers new models of learning opportunities for individuals at any time [6]. Despite the fact that over three and a half million students are estimated to be currently enrolled in e-learning classes, there is limited information available regarding its level of flexibility, distribution, and openness [7]. E-learning has a positive impact on the academic achievements of students, as it reduces costs, saves time, increases accessibility of education, and enhances academic performance [8]. However, there are challenges in providing e-learning education in universities, such as the lack of financial and physical resources to supply the necessary computer systems and equipment, which are major obstacles to its development [9, 10]. E-learning based education infrastructures encompasses technological tools, systems and structures that facilitate proper access to e-learning services [11]. Furthermore, the results of various studies have demonstrated that the most significant obstacles to e-learning are unsuitable organizational structures, cultural and environmental challenges, as well as negative public perceptions [12, 13]. During the COVID-19 pandemic, several studies have suggested that learners were not adequately prepared for e-learning and expressed dissatisfaction with the method [14–16]. According to a study, medical students expressed dissatisfaction with the use of e-learning technology when compared to in-person classrooms, citing feelings of isolation and disconnection from their peers and instructors [17]. García-González et al. (2021) reported that a significant majority of nursing students experienced emotional impacts such as elevated levels of anxiety due to the shift to online learning during the COVID-19 outbreak [18]. Results from a study of nursing students indicated that 61.6% of students had negative attitudes to e-learning [16]. However, the results of another study indicated that nursing students had a positive e-learning experience [19]. Elzainy and colleagues indicated that E-learning was highly beneficial for competent educators as it decreased the need for in-class attendance, compensated for the suspension of face-to-face teaching, and provided more opportunities for open discussions [20]. E-learning has been shown to be effective and valuable in nursing education, as it actively engages learners in the learning process and facilitates self-directed learning. The continuous presentation of teaching materials can improve student motivation, satisfaction, and enjoyment compared to other methods of instruction [21].

Nursing students in low-income countries often hold negative attitudes towards online learning, primarily due to the high cost of internet connections and slow internet speeds [14]. Despite the importance of understanding nursing e-learning based education during the COVID-19 pandemic from a qualitative perspective, the majority of studies conducted have been quantitative, with few qualitative studies exploring this issue [15]. Qualitative research provides detailed descriptions of participants’ experiences and feelings, emphasizing subjective and diverse perspectives to gain an in-depth understanding of a social issue or problem [22]. Qualitative content analysis is a widely used method in nursing science that can be applied to a variety of contexts and data sources [23]. Qualitative content analysis systematically codes and classifies text to uncover patterns and themes, generating knowledge and understanding of the phenomenon being studied beyond simple word counting [24].

This study aims to describe challenges and facilitators of e-learning in nursing education during the COVID-19 pandemic from the perspective of those involved, using a qualitative approach.

## Methods

### Study design

The present research is a qualitative study that utilized the content analysis method, employing a conventional approach and following the qualitative analysis steps outlined by Graneheim and Lundman [25]. Conventional content analysis is the most commonly used method for analysis in studies aimed at describing the properties of a phenomena. It helps the researcher to understand social phenomenon in a subjective yet scientific manner [24].

### The participants and settings

In this study, the participants comprised of students, teachers, and staff of the educational unit at Nasibeh Faculty of Nursing and Midwifery in Sari, Iran. A purposeful sampling method was employed to collect data. The purposeful diversity in the sample selection was intended to facilitate the wider applicability of the research results to its intended audience [26]. The researchers approached potential participants and invited them to participate in the study. Those who expressed interest were provided with detailed information about the study. The participants were not required to disclose their names and provided written informed consent. The sample consisted of eight nursing students from different semesters, six teachers with varying levels of academic experience, and two educational unit staff members with diverse work positions (Table 1).

**Table 1:** Demographics characteristics of the Participants

Code	Age (yrs.)	Gender	Job	Position /Semester
1	22	Woman	Student	3rd Semester three
2	22	Woman	Student	4th Semester
3	38	Woman	Faculty member	Assistant Professor
4	20	Man	Student	2nd Semester
5	25	Woman	Student	8th Semester
6	55	Woman	Faculty member	Assistant Professor
7	60	Woman	Faculty member	Associate Professor
8	22	Woman	Student	5th Semester
9	21	Woman	Student	3rd Semester
10	35	Woman	Educational expert	Official employee
11	55	Man	Faculty member	Associate Professor
12	22	Man	Student	7th Semester
13	40	Man	Information Technology Official	Official employee
14	22	Man	Student	6th Semester
15	46	Woman	Faculty member	Assistant Professor
16	42	Man	Faculty member	Assistant Professor

### Data collection

Data was collected through semi-structured interviews, which were conducted in a private room at the faculty to ensure the privacy of the participants, while adhering to health and safety protocols for COVID-19 prevention. The interviews and data collection period commenced from November 2020 and continued until February 2021, with the aim of achieving theoretical saturation. The term “saturation” refers to the point at which the data collected contains sufficient information to address the research questions at hand [27]. Upon obtaining verbal consent and ensuring confidentiality, face-to-face interviews were conducted using open-ended questions. The interviews were recorded with the participants’ consent. They were asked questions such as: “Would you please tell me your experiences during the providing e-learning in the faculty?“ “Would you like to describe the experiences regarding the problems of e-learning education?“ “What are the supporters of e-learning?“ Throughout the interview, emphasis was placed on eliciting the participant’s personal experiences and perspectives related to their e-learning education. To gain a deeper understanding of the participants’ experiences and perspectives, follow-up and probing questions were asked based on the data provided during the interviews. The English version of interview guide, which contains details related to the interview questions, is provided in the supplementary file. The researcher aimed to be an attentive listener throughout the interview process. The interviews typically lasted between 30 and 50 minutes.

### Data analysis

Data analysis involved creating an immediate summary of each interview, followed by a thorough review of the recorded interviews and transcribed recordings. The qualitative approach developed by Granheim and Lundman was employed for data analysis [25]. The data analysis process involved several steps. First, the researchers read through all the transcripts to gain a comprehensive understanding of the data. Units of meaning, which could be a word, sentence, or an entire paragraph that pertained to the study question (What are the challenges and facilitators of nursing student e-learning during the COVID-19 pandemic?), were identified and marked. These units of meaning were then condensed into a description of their manifest content and an interpretation of their latent content. Subsequently, the researchers abstracted sub-themes from the descriptions and interpretations. Through a process of reflection and discussion, the researchers agreed on a number of sub-themes and identified relevant headings that would unify the sub-themes into themes [28]. The transcripts were reviewed multiple times to enhance understanding. Semantic units, such as paragraphs, sentences, or words, were identified and summarized based on their meaning. Similar codes were compared and contrasted, and then grouped into more abstract categories labeled with specific headings. Finally, the categories were compared to one another to identify overarching themes that encapsulated the data.

### Trustworthiness

To ensure the reliability of the data, an expert with qualitative research experience reviewed all the codes, subcategories, and categories, as recommended by Guba and Lincoln [29]. The researchers aimed to enhance the credibility of the study by selecting participants with diverse experiences, establishing rapport and engaging in sufficient interaction, collecting valid information, and validating the findings with the participants. Additionally, the researchers maintained continuous engagement with the data throughout the analysis process. The findings were presented to the participants and a group of experts for verification, and any discrepancies were addressed and resolved.

For dependability, the audio recording from the interviews was transcribed verbatim into a text file. The process of the transcribing and coding was done independently by two researchers, and then the results were compared and a final decision was made.

To enhance confirmability of the findings, the research team made a concerted effort to avoid any preconceived notions or biases during the analysis process. The coding process of the interview transcripts was finalized through consultation and consensus among the research team members.

To enhance the transferability of the study findings, the researchers provided a detailed and comprehensive description of the research findings, which may be applicable to other fields. The presentation of the participants’ statements was faithful to their original wording. The use of convenience sampling with maximum diversity contributed to the transferability of the study results. Other researchers may be able to perceive the new concepts and insights that emerged from the study, which could contribute to nursing education and related fields.

## Results

The study included 16 participants who had experience with nursing e-learning during the COVID-19 pandemic. The data were categorized into two categories: e-learning challenges and e-learning facilitators. The e-learning challenges category consisted of five subcategories, including inexperienced teachers, Ineffective learning, Academic cheating, System problems, and Inappropriate evaluation (Table 2). The e-learning facilitators category included two subcategories, improving education and Promoting online exams (Table 3).

**Table 2:** Category of e-learning education challenge, its subcategories and condensed codes

Category	Subcategories	Condensed codes
Challenges of e-learning	Inexperienced teachers	• Incapable to use Camtasia software • No e-learning training experience • High and bulky loading of files compared to the number of units • Concurrently loading several files at the same time, especially near the end of the semester exam • Uncertain scheduling of sending files • Not using the forum option in the system
	Ineffective learning	• Some teachers use English content in the files • Failure to perceive some basic science courses’ content • Non-class attendance compulsory • Not continuing learning by the student • Reduced communication between students and teachers • Teacher-centered teaching • Education without using educational aids
	Academic cheating	• Copying assignments and sending them to professor •a Variety of cheating in online education • Forming a cheating group in the class
	System problems	• Some students not accessing the Internet • Lack of hardware and software facilities at the beginning of the pandemic • Low internet speed • The problem of uploading and downloading large files • Structural problems of Navid software
	Inappropriate evaluation	•Lack of feedback from student learning • Unrealistic evaluation of students • Reduced evaluation quality due to the inability to send assignments • Limited variety of test questions

**Table 3:** Category of facilitators of e-learning, its subcategories and condensed codes

Category	Subcategories	Condensed codes
Facilitators of e-learning	Improving education	•Enhancing teachers’ skills in using e-learning facilities • Integrating several methods for teaching • Using specific schedule to upload files • Holding online question-answer session • Encouraging students to be more active
	Promoting online exam	• Providing a better infrastructure for exams • increasing the number of online exams • Uploading more assignments by teachers •Performing standard tests under suitable conditions

### Challenges of E-learning Education

The first category that emerged from the analysis of the participants’ statements on their e-learning experience during the COVID-19 pandemic was “E-learning challenges,“ which consisted of the following five subcategories:

### Inexperienced teachers

The participants’ statements indicated that some teachers lacked sufficient experience with e-learning during the COVID-19 pandemic. Specifically, the participants noted that these teachers faced challenges with using Camtasia software (an interactive online learning video software [30]), had limited experience with e-learning-based education, struggled with uploading large files of educational materials, had difficulty uploading several files simultaneously, did not adhere to a schedule for uploading files, and did not utilize the forum session.

Participants shared their experience as follows:

“In the first course of online training, the teachers uploaded a large amount of files close to the exam time…” (Participant 1).

One of the teachers said: “At the beginning, it was very difficult for me, it was all trial and error to record audio and video in Camtasia software.” (Participant 3).

### Ineffective learning

According to the participants’ statements, “Ineffective learning,“ particularly in vocational training, was another subcategory of e-learning challenges. Participants identified problems such as the use of English language in educational materials, difficulty in understanding the educational content for some students, high levels of student absenteeism in online classrooms, irregular learning patterns by some students, poor interaction among students and with the teacher, teacher-centered teaching, and a lack of use of teaching aids. Participants shared their experiences in this regard:

“In the Navid system (a software for uploading educational materials), you cannot ask your own questions directly; you can ask the teacher through sending a message, but I prefer not to ask”. (Participant 5)

“I’m not fond of the online classroom because I’m not in contact with the students in person. It’s not enjoyable for me at all. As I can’t see the students’ faces, I can’t get the due feedback from my class.” (Participant 6).

“Lots of the students are from the rural areas and may not have access to the Internet, and some said that they do not have a laptop or a computer, and the Navid system cannot be loaded on their mobile phones.” (Participant 9).

### Academic cheating

Another subcategory of e-learning challenges was “Academic cheating.“ Participants noted instances such as copying assignments and submitting them to teachers, the use of various cheating methods in e-learning education, and the formation of cheating groups in online classes. Participants shared their experiences as follows:

“I feel bad when some students cheat during exams or they send us duplicated assignments. “(Participant 11).

“Usually in the online exam, there is room for some students to cheat despite the teachers trying to control it.” (Participant 10).

### System problems

Participants also discussed system-related problems, such as issues with internet access, a lack of sufficient hardware and software facilities at the onset of the pandemic, slow internet speeds, difficulties with uploading and downloading large educational files, and challenges with working with Navid software. Participants shared their experiences as follows:

“E-learning education costs a lot to upload and download large files for the teachers and the students” (participant 14).

“Considering that the main server is in the headquarters of the university and we do not access it, we could not solve the problems of disconnections immediately.” (Participant 13).

### Inappropriate evaluation

Based on the participants’ experiences, an inappropriate educational evaluation was identified as another e-learning challenge. Although educational evaluation is an important component of the learning process, participants noted that it did not have sufficient credibility in the context of e-learning education. Participants shared their experiences as follows:

“When I find out about students’ cheating, I don’t know what may happen to their educational evaluation matter.” (Participant 16).

“Considering that some students pass the exam by cheating, the rights of the students who painstakingly handwrite and studied the files are violated.” (Participant 2).

### E-learning education facilitators

Another main theme that emerged from this study was e-learning facilitators, which included two subcategories: improving education and promoting online exams.

### Improving education

To improve Education, participants identified several measures, such as enhancing teachers’ skills in using e-learning facilities, integrating multiple teaching methods, adhering to a specific schedule for uploading files, retraining teachers to work optimally with the software, holding online question-and-answer sessions, encouraging student participation, and increasing teachers’ access to online webcams. Participants shared their experiences as follows:

“I was the advisor of the first semester students, and on a daily routine, I asked them about the study hours through the WhatsApp group, which encouraged competition and increased their studies.” (Participant 15).

“We had a teacher who presented the lesson to us through files, both audio and video in the Navid system, and he solved our course related problems through WhatsApp.” (participant 12).

From the teachers’ perspective, online courses were considered more effective for learning because they were similar to in-person classes. The active presence of the students, the question asking and answering potential, the possibility of seeing the teacher while presenting the material, creating more motivation and attention in learning are some of the merits of online education.

Participant number eight stated that:” In online education, seeing the teacher in person can be an encouragement for the student to study.“

Or participant number two said:” In online class, the communication problem with the student is somewhat solved.”

Participant number four stated so:” For online classes, it is better if the teachers hold the classes after five pm to have better interaction with the students.”

### Improving online exam

Participants discussed potential solutions to ensure more valid exams. They suggested improving infrastructure, increasing the number of online examinations, having teachers upload more assignments, and conducting standardized tests under appropriate conditions. The participants provided the following accounts of their experiences:

Participant number seven said:” At the end of the class, I usually asked three to four questions from the lesson so that to come up with good assessment “.

Participant number three also stated: “It’s better to adjust the exam time and the answering duration.”

Participant number 13 said: “we predicted all the possible solutions for online exam, for instance we determined a specific time for each question and the exam questions their answer were randomly presented to reach more desirable evaluation.”

In the present study, the students mentioned some advantages accrued through e-learning based education, such as the lack of attendance in the physical classroom, being free to choose the time and type of the course for studying, easy access to the educational material which had been saved in the software system, saving time and money, not requiring to hold compensatory.

## Discussion

The study aimed to identify the challenges and facilitators of e-learning in the nursing faculty, as well as to explore potential solutions to improve the effectiveness of e-learning. The participants’ insights and experiences provided valuable insights into the practical aspects of e-learning and highlighted the importance of adapting to new modes of education during a crisis.

### Inexperienced Teachers

The findings showed that some teachers had insufficient experience with e-learning based education. Leigh and colleagues (2020), reported that nursing schools used unfamiliar technology of e-learning to present theoretical lessons and the COVID-19 pandemic has forced educators to rapidly adapt to digital technologies in order to continue to provide high-quality nursing education remotely. This has been a challenging process for many educators who may have been less familiar with e-learning platforms and digital tools [31]. In the study by Nabolsi and others (2021), the majority of the participants had their first experience in e-learning and they lack of technical skills to manage e-learning effectively, and they suggested that ongoing training and support can help faculty feel more comfortable with online teaching and improve the quality of their instruction [32]. Additionally, a study by Shafiei and colleagues (2019) identified teachers’ incapability to apply e-learning, negative attitudes towards e-learning, and a lack of desirable interaction between teachers and students as challenges of e-learning in Iran [7].

### Ineffective learning

Another challenge of e-learning was ineffective learning, which was associated with poor interaction and communication between teachers and students, a lack of opportunities to discuss and resolve ambiguity during teaching sessions, teacher-centered education, and a lack of use of educational aids.

While e-learning can be effective in improving nursing knowledge and skills, it may not be suitable for all types of learning or all learners. Some learners may prefer more traditional forms of education, such as classroom-based instruction, or may struggle with the technology or lack of personal interaction. Therefore, it is important for educators and institutions to carefully consider the use of e-learning and to provide support and resources to ensure that learners can fully engage with the learning experience [33]. Magner and others (2014), also noted that providing educational feedback to the students can increase the quality of learning by creating motivation and situational interest [34]. Nursing educators should provide clear guidance and support for online learning, and that they prioritize opportunities for social interaction and support to combat feelings of isolation and disconnection [35]. Besides, Inability to concentrate and to avoid e-learning activities can negatively affect academic progress [36]. It is necessary that educators and institutions prioritize the provision of resources and support to enable students to fully engage with e-learning, while also exploring hybrid models of education to meet the diverse needs and preferences of students [4]. In addition, the findings of a systematic review study suggested that e-learning tools may become increasingly important in medical education even after the pandemic subsides. Students should have access to high-quality online resources and interactive digital platforms to support their learning needs [37]. However, the transition to e-learning required students and faculty members to adapt to new teaching methods and strategies, which required time and effort [20].

### Academic cheating

Another finding mentioned by the participants was academic cheating, which can prevent individuals from fully engaging in the learning process and developing necessary knowledge and skills. Therefore, universities need to develop solutions to ensure the security of online tests and prevent academic dishonesty [38]. The lack of direct supervision and monitoring of students in online exams can make it easier for students to cheat by accessing unauthorized resources or collaborating with others. Some strategies such as using plagiarism detection software, designing exams that require critical thinking and application of knowledge, and implementing remote proctoring to monitor students during online exams, can be used to address this issue. These strategies can help promote academic integrity and ensure that students are evaluated fairly and accurately [39]. Holden and colleagues (2021) emphasized in their systematic review study that the application of artificial intelligence in developing online tests was helpful in reducing cheating during exams [40].

### System problems

System problems was a subcategory of e-learning challenges. As the study of Koohpayehzadeh and others (2017) suggested, the development of the technological factors such as the infrastructure, network, software and hardware are essential for e-learning based education [41]. The need for access to appropriate technology and internet connectivity, can be a barrier for some students [42]. Currently, more than 26% universities of medical sciences in Iran lack the necessary hardware and software infrastructures to implement e-learning based education [43]. Additionally, research by Özkan and colleagues (2021) identified poor internet speed and frequent power outages in rural areas as the biggest obstacle against distance learning for nursing students [44].

### Inappropriate evaluation

Inappropriate evaluation was another finding of the study. Sadeghi Mahali and colleagues (2022) also emphasized that one of the most significant challenges of e-learning education was the problem of educational evaluation [45]. Similarly, in a study by Yassini (2015), the evaluation of e-learning courses was reported as ineffective from the students’ perspective [46]. Other research findings have also revealed that student evaluation was a challenge in online medical education during the COVID-19 pandemic in Saudi Arabia [47]. The emergence of the COVID-19 pandemic has brought about changes in how learners are evaluated in relation to their level of learning [48]. Furthermore, just like with all educational activities, ensuring the quality of e-learning programs is crucial and considered an integral part of e-learning [47]. According to a study, students who regularly participated in online discussion forums performed significantly better than those who did not. Furthermore, there is a positive correlation between the amount of time spent on the e-learning platform and student performance [49]. This information can be used to improve the evaluation of students’ academic progress.

In addition to the challenges, this study also identified facilitators for promoting e-learning education, which included two subcategories: Improving education and promoting online exams.

### Improving education

According to the participants, enhancing teacher skills through retraining, integrating multiple teaching methods, and organizing question-and-answer sessions are essential to improve e-learning based education. E-learning technologies have been found to enhance interactive teaching and learning processes [50], providing a high-quality and sustainable educational infrastructure that fosters student participation and cooperation with teachers. Furthermore, the change in the teaching process during the COVID-19 pandemic has provided an opportunity for teachers to develop their skills in using e-learning facilities [1, 51]. Despite the benefits of e-learning based education, it is generally not a substitute for traditional education methods, but can be used as a complementary method [52]. To encourage group discussions and critical thinking, teachers may use the Socratic Method in e-learning classrooms. This method involves purposefully asking and answering questions consecutively [31]. However, as students and teachers had limited e-learning experience before the COVID-19 pandemic, they generally preferred face-to-face sessions, and the mandatory shift to e-learning education occurred suddenly, causing pressure, uncertainty, and anxiety [52]. Amidst the COVID-19 outbreak, teachers faced numerous challenges while working with educational technologies, such as a lack of awareness of new e-learning educational methods and ways to minimize student distractions [53]. The main concerns of faculty teachers and students during the COVID-19 pandemic in e-learning education were the inability to deliver practical courses and internships, ensuring accurate exams and preventing cheating during online tests, and reducing social interactions [54]. However, e-learning technology has provided an environment for independent and self-directed learning, the ability to learn anywhere at any time, collaborative teaching and learning, and efficient evaluation and feedback provision for lessons [55].

### Promoting online exam

Over the past three years, the practice of online evaluation has significantly increased, especially since the COVID-19 pandemic [56]. Online exams have become an integral part of e-learning solutions for true and fair assessment of student achievement [57]. Online exams offer potential benefits, such as increased accessibility and cost-effectiveness, leading to greater student engagement. They can also provide objective evaluation, timely feedback, and comprehensive assessment. However, challenges such as technical issues, cheating, and infrastructure require further research to optimize online exams for promoting student learning and performance [57].

### Study limitations

The small sample size and recruitment of participants from a single nursing school limit the generalizability of the results to other nursing schools and settings.

## Conclusion

During the COVID-19 pandemic, the use of e-learning became a necessity in most nursing schools worldwide due to the suspension of in-person teaching. However, this shift to e-learning brought its own set of challenges and opportunities. Nursing schools that adopt e-learning need to be aware of the potential challenges and find ways to address them. They should also strengthen the facilitators who use this method of education. To improve e-learning education in nursing faculties, programs that increase the knowledge of nursing teachers to work with e-learning related software and hardware should be developed. This can provide a more effective learning environment for nursing students and lead to better evaluation and testing of their progress. Furthermore, providing the necessary infrastructure and equipment, such as appropriate systems and high-speed internet, is essential to ensure that students have a seamless e-learning experience. By addressing the challenges and optimizing the facilitators of e-learning, nursing schools can successfully adapt to this new mode of education in the post-pandemic era.

### Electronic supplementary material

Below is the link to the electronic supplementary material.

Supplementary Material 1

## Data Availability

The data supporting the findings of this study are available upon reasonable request from the corresponding author, [T.Y.]. However, the data cannot be made publicly available due to concerns regarding the compromise of participants’ privacy.
