# Supplementary material for: Exploring challenges and facilitators to E-learning based Education of nursing students during Covid-19 pandemic: a qualitative study
Source: BMC Nurs. 2023 Aug 22;22:278. doi: 10.1186/s12912-023-01430-6 (PMC10463958; doi:10.1186/s12912-023-01430-6)
Supplement: Supplementary file 1 — Supplementary Material 1 [file 12912_2023_1430_MOESM1_ESM.docx]

**Interview guide for the study of “Exploring challenges and Facilitators to E-learning based Education of Nursing students during COVID-19 Pandemic: A Qualitative Study”**

**Introduction:**

- The researcher introduces herself and explains the purpose of the interview, which is to explore the experiences of E-learning based Education of Nursing students during the Covid-19 pandemic.

- Explain that the interview will take approximately 30-45 minutes.

- Obtain informed consent from the interviewee.

**Background questions:**

- Would you please tell me your experiences during the providing e-learning in the faculty of Nursing?

- What were your expectations going into e-learning?

- How did you feel about the transition to e-learning?

**Challenges with e-learning:**

- Would you like to describe the experiences regarding the problems of e-learning education?

- How did you overcome those problems?

- Were there any specific areas of e-learning that were particularly difficult for you?

**Facilitators of e-learning:**

- What helped you succeed in e-learning?

- Were there any particular resources or tools that you found helpful?

- What are the supporters of e-learning?

**Follow-up and probing questions:**

- How do you feel e-learning has impacted your learning and professional development?

- Do you feel you have gained the same level of knowledge and skills as you would have with in-person learning?

**Closing:**

- Do you have any additional thoughts or comments you would like to share on the topic of E-learning based education of nursing students during the Covid-19 pandemic?

- Thank the interviewee for the time and participation.
